# Molecular Mechanism Underlying Role of the XBP1s in Cardiovascular Diseases

**DOI:** 10.3390/jcdd9120459

**Published:** 2022-12-14

**Authors:** Shu Liu, Hong Ding, Yongnan Li, Xiaowei Zhang

**Affiliations:** Department of Cardiology, Lanzhou University Second Hospital, Lanzhou University, Lanzhou 730030, China

**Keywords:** spliced X-box binding protein-1, hypertension, cardiac hypertrophy, heart failure, cardiovascular diseases

## Abstract

Spliced X-box binding protein-1 (XBP1s) is a protein that belongs to the cAMP-response element-binding (CREB)/activating transcription factor (ATF) b-ZIP family with a basic-region leucine zipper (bZIP). There is mounting evidence to suggest that XBP1s performs a critical function in a range of different cardiovascular diseases (CVDs), indicating that it is necessary to gain a comprehensive knowledge of the processes involved in XBP1s in various disorders to make progress in research and clinical therapy. In this research, we provide a summary of the functions that XBP1s performs in the onset and advancement of CVDs such as atherosclerosis, hypertension, cardiac hypertrophy, and heart failure. Furthermore, we discuss XBP1s as a novel therapeutic target for CVDs.

## 1. Introduction

Since the 20th century, the prevalence and incidence of cardiovascular diseases (CVDs) have increased exponentially worldwide, with devastating medical and economic consequences. Approximately 17.9 million people die from CVDs worldwide every year [1]. The global increase in cardiovascular disease requires a thorough understanding of the pathophysiological molecular mechanisms of disease occurrence. Over centuries of research into the pathophysiology of cardiovascular diseases, we have identified several classes of pathological processes and metabolic systems that are critical to human health. Dysfunctional endoplasmic reticulum (ER) stress and unfolded protein response (UPR), characterized by the obstruction of the production of key ER proteins in the body and an increase in the accumulation of potentially toxic misfolded proteins, are typical examples of the pathophysiological conditions found in patients with cardiac, vascular, and metabolic diseases [2].

**Figure 1:**
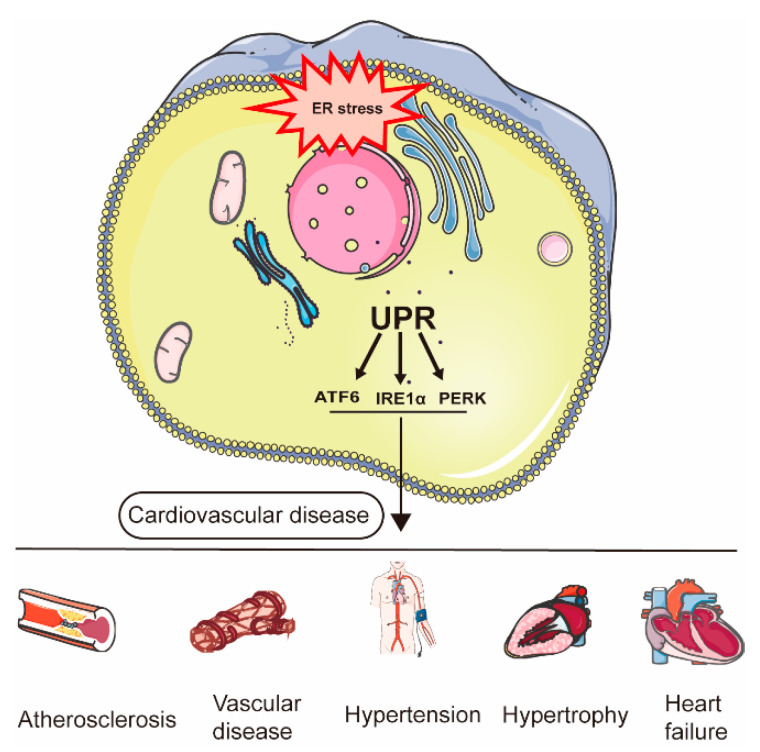
The mechanism of ER stress and UPR in cardiovascular disease. ER stress: endoplasmic reticulum stress; UPR: unfolded protein response. IRE1a: inositol-requiring kinase 1; ATF6: activating transcription factor 6; PERK: protein kinase-like ER kinase.

The ER is a membranous organelle involved in the production and maturation of proteins and is crucial for the folding, maturation, and post-translational modification of proteins [3]. Cellular stress caused by physiological or pathological stimuli contributes to the accumulation of misfolded or unfolded proteins in the ER, which imposes a load on the ER protein-folding mechanism, thus overwhelming its capacity, a state known as ER stress (ERS) [4]. To guarantee that the capacity for protein folding is balanced with the required level, cells have evolved an adaptive signal transduction pathway that transduces signals from the ER to the nucleus, called the UPR, which attempts to restore ER homeostatic balance and alleviate or reduce stress [5]. This UPR pathway has salient and unique features [2]. For example, it can induce translational attenuation, which then lowers the number of nascent proteins that are translocated into the ER. Furthermore, the UPR is capable of transcriptionally activating the genes that encode ER chaperones and enzymes to restore the expression of folding-defective proteins. Last but not least, the UPR works through the induction of the ER-associated degradation (ERAD) components and degradation of misfolded or unfolded proteins. A compensational reaction to ER stress mainly involves the activation of three signaling pathways by the following sensor proteins: Inositol-requiring kinase 1 (IRE1a), activating transcription factor 6 (ATF6), and double-stranded RNA-activated protein kinase-like ER kinase (PERK) [6]. Under normal conditions, glucose-regulating protein 78 (GRP78), the ER chaperone, binds to the luminal domains of these sensors and keeps them inactive. Under stressed conditions, GRP78 dissociates from IRE1α, PERK, and ATF6 and allows UPR activation, which occurs in a variety of cardiovascular diseases (Figure 1). These proteins collaborate in a complementary manner to bring the system of unfolded proteins/chaperones into a state of homeostasis in the ER. Differential roles of the individual UPR transducers have been proposed [7]. PERK is a kinase that is responsible for the phosphorylation of the downstream eukaryotic translation initiation factor 2α (eIF2α) to induce an immediate adaptive response to ER stress [8]. eIF2α can further improve the capability of the ER to fold proteins by processing the translation of the mRNA encoding factor 4 (ATF4) [9]. As one of the major signaling pathways of ER stress, ATF6 plays a significant role in regulating gene coding. Once ATF6 has translocated to the Golgi, it is cleaved by the proteasomes at sites 1 and 2. The cleaved fragment of ATF6 eventually enters the nucleus to regulate the genes encoding the ERAD components and X-box binding protein-1(XBP1) [10]. The IRE1α pathway is a branch of the UPR that is considered to be highly conserved [11]. IRE1α is an ER transmembrane protein that functions as both a protein kinase and an endoribonuclease (RNase). In the presence of ER stress, IRE1α dimerizes and undergoes trans-autophosphorylation via the cytoplasmic kinase domain. Upon activation of the UPR, activated IRE1α processes the XBP1 mRNA to generate a spliced form of the transcription factor XBP1 (XBP1s) that is both functional and highly active by the removal of a 26-base intron from the initial mRNA in an unconventional splicing reaction [12]. Abundant studies have shown that XBP1s is engaged in a variety of human CVDs. Targeting XBP1s is considered a promising treatment strategy for CVDs. Regulation of this target by inhibition or activation may bring different clinical benefits depending on the CVDs status. However, this is not a comprehensive review. In the disease conditions described below, we focus on related CVDs, in which the regulation of the XBP1s pathway may lead to the development of new therapies. Therefore, we review a variety of cellular functions of XBP1s and the molecular mechanism underlying the regulatory role of XBP1s in CVDs.

## 2. XBP1s Structure and Cellular Function

**Figure 2:**
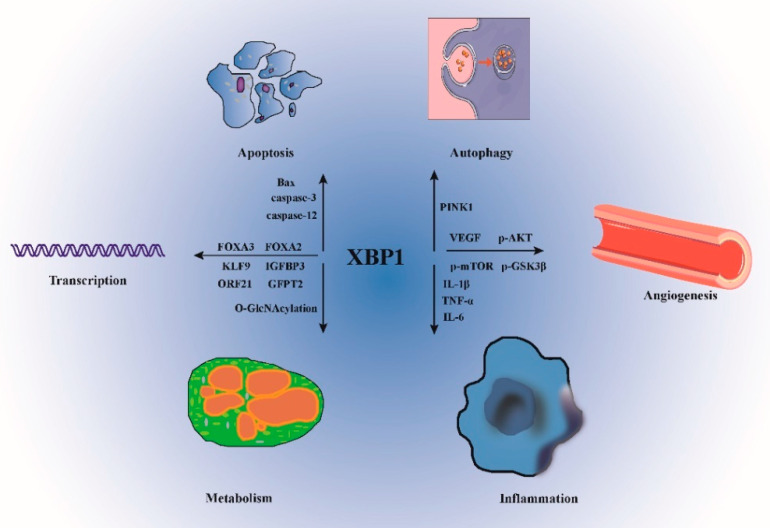
Research into the functions of XBP1s. XBP1s plays an important role in cardiovascular diseases by interacting with various signaling pathways. KLF9: Krüppel-like factor 9; FOXA3: Forkhead Box A3; IGFBP3: insulin-like growth factor binding protein-3; FOXA2: Forkhead Box A2; GFPT2: glutamine-fructose-6-phosphate transaminase 2.

XBP1s belongs to the cAMP-response element-binding (CREB)/activating transcription factor (ATF) b-ZIP family with a basic-region leucine zipper (bZIP) [13]. XBP1s is composed of the original DNA binding domain at the amino terminal as well as a transactivation domain at the C-terminal and performs the role of a stress-inducible powerful transcriptional factor by binding to the UPR element and therefore modulating the expression of the UPR target genes that encode proteins. XBP1s moves to the nucleus in response to IRE1 activation, where it then stimulates the upregulation of its target genes that encode ER-associated degradation (ERAD) components and ER chaperones, hence leading to an enhancement of the ER’s ability to fold newly generated proteins and degradation of proteins that are either misfolded or unfolded [14]. Thus, strategies regulating the proper function of XBP1s to reduce ER stress might provide attractive pathways for the development of innovative therapeutic approaches for CVDs. Notably, there is an increasing body of data to show that XBP1s may function as a modulator in CVDs, particularly in atherosclerosis [15], myocardial ischemia/reperfusion injury [16], and cardiac hypertrophy [17]. Interestingly, in some cardiovascular diseases, the involvement of XBP1s is also independent of ERS and UPR activation [18]. Furthermore, XBP1s participates in transcriptional regulation, apoptosis, and oxidative stress (Table 1 and Figure 2). However, a detailed specification is necessary to provide valuable insights into the mechanisms behind XBP1s’ involvement in the development of CVDs, and to facilitate the development of an optimal CVD-related treatment that will reverse the progression of the illness and eventually result in the patient being cured.

## 3. The Role of XBP1s in Smooth Muscle Cells

**Figure 3:**
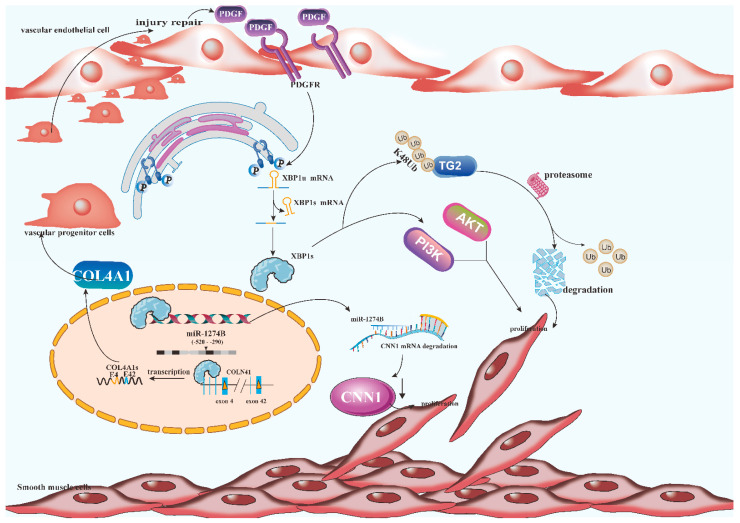
Schematic representation of the critical molecular mechanism by which XBP1s regulates smooth muscle cells proliferation and neointima formation in smooth muscle cells. PDGF may be released in areas of injury caused by vascular injury. The released PDGF binds to PDGFR on smooth muscle cells, causing PDGFR to interact with IRE1 and activate phosphorylation of IRE1 and splicing of XBP1 mRNA. XBP1s directly linked to the region 520 to 290 of the mir-1274B promoter, activated miR-1274B transcription, targeted and degraded CNN1 mRNA, resulting in decreased CNN1 protein and decreased SMC proliferation. The XBP1s protein directs the transcription of COL4A1 gene by binding to the enhancer in exons 4 and 42 of COL4A1 gene. COL4A1s acts as a paracrine cytokine to mobilize vascular progenitor cells (VPCs) to the site of injury, thereby promoting wound healing or disease improvement. TG2 induces β-catenin and PDGF signaling in VSMCs, which in turn enhances the proliferation, migration, and phenotypic switching ability of these cells. Cells lacking XBP1s can increase the proteasomal breakdown of TG2 by promoting K48 polyubiquitination. PDGF: Platelet-derived growth factor, CNN1: calponin h1, COL4A1: type IV collagen alpha 1. TG2: Transglutaminase 2.

Smooth muscle cells (SMCs) are the principal cell constituents of the vessel wall and are responsible for maintaining blood vessel pressure and tone [50]. In recent studies, it has been shown that XBP1 splicing may be triggered in a way that is independent of ER stress by physiological triggers which include vascular endothelial cell growth factor (VEGF). Platelet-derived growth factor (PDGF) is a mitogenic factor that is extensively recognized for its role in the proliferation of mature SMCs [51]. PDGF/PDGFR binding activates several signaling pathways, including mitogen-activated protein kinases [52], PI3K/Akt [53], and basic fibroblast growth factor (bFGF) [54]. PDGF may be released in the injured area triggered by vascular injury. The released PDGF may then bind to PDGFR on SMCs, causing PDGFR to interact with IRE1, and in this way, both the phosphorylation of IRE1 and the splicing of XBP1 mRNA are activated. In contrast, calponin h1 (CNN1) overexpression is known to suppress SMC proliferation and neointima formation. MiR-1274B secreted into culture medium performs the role of a paracrine factor to downmodulate the CNN1 mRNA in neighboring cells (Figure 3). Using a chromatin immunoprecipitation test, it has been discovered that XBP1s is directly attached to the domain of the mir-1274B promoter spanning from positions 520 to 290. Studies have shown that XBP1s not only activates the PI3K/Akt pathway, which increases the migration of SMCs, but also miR-1274B transcription, which targets and degrades CNN1 mRNA, resulting in a decrease in the CNN1 protein and SMC proliferation [55]. By binding to the enhancers included within exons 4 and 42 of the type IV collagen alpha 1(COL4A1) gene, the XBP1s protein is capable of directing the transcription of the COL4A1 and COL4A2 genes. To get rid of the internal sequence, the resultant COL4A1 mRNA could undergo an unconventional splicing process that is mediated by IRE1α between exons 4 and 42. Alternatively, the XBP1s attached to exons 4 and 42 might combine into a dimer, which would bring the two exons closer together and direct the transcription of RNA polymerase from exon 4 to exon 42, bypassing the internal sections. Both mechanisms result in the production of a shorter, more soluble isoform of COL4A1s, which performs the role of a paracrine cytokine to mobilize vascular progenitor cells (VPCs) to the wounded sites, thus contributing to the healing of injuries or the improvement of illnesses [56]. In arterial diseases, transglutaminase 2(TG2) induces β-catenin- and PDGF- signaling in VSMCs, which in turn enhances the proliferative, migratory, and phenotypic switching capacity of these cells. We observed that cells lacking XBP1s could increase the proteasomal breakdown of TG2 by promoting K48 polyubiquitylation, particularly in comparison to SUMOylation. The process of protein SUMOylation may help proteins become more stable, partly because it inhibits ubiquitination at lysine residues, and partly because proteins that have been SUMOylated exhibit a very unique pattern of surface charge distribution compared to ubiquitinated proteins [57].

## 4. The Role of XBP1s in Ischemia/Reperfusion Injury and Atherosclerosis

Local ischemia occurs when a local blood artery is occluded, which may happen as a consequence of an injury, atherosclerosis, or thrombosis, leading to cell death and impaired function in the ischemic organ. Angiogenesis is one of the processes that helps restore blood circulation in ischemic tissues. A significant aspect of this process involves VEGF mediated endothelial cell (EC) migration and proliferation. The splicing of XBP1s mRNA has a crucial function in VEGF signaling and leads to the proliferation and angiogenesis of endothelial cells in ischemic tissues [58]. Numerous lines of evidence also have indicated that ischemia stimulates the activation of stress response pathways, which may help prevent damage to tissues. Furthermore, XBP1 mRNA is spliced as a response to hypoxia, which also results in an elevation in the level of XBP1s and induces GRP94 and GRP78 [59]. GRP78 is a direct transcriptional target of the UPR-induced transcription factor XBP1s [60]. The majority of acute coronary syndromes are caused by the destabilization and rupture of atherosclerotic plaques. Macrophages and the secretory products that they produce have a significant impact on the destabilization of plaque [61]. Molsidomine, a donor of nitric oxide (NO), was used in previous research to treat atherosclerosis in rabbits, which resulted in the clearance of subendothelial macrophages and the formation of plaques primarily composed of collagen fibrosis and SMCs. Hyperphosphorylation of eIF2a, suppression of de novo protein synthesis and splicing of XBP1 mRNA may serve as the mechanism behind an NO donor’s ability to selectively remove macrophages from atherosclerotic plaques [62]. Toll-like receptors (TLRs) specifically activate XBP1s via NOX2 NADPH oxidase, which is required for the optimal and sustained production of proinflammatory cytokines in macrophages [63].

ECs are essential biological constituents of blood vessels, performing the role of barriers between blood and tissues that are selectively permeable [6]. It is hypothesized that risk factors cause EC to undergo apoptosis, which then results in the disruption or malfunction of the intact endothelium monolayer. This results in the accumulation of lipids, the adhesion of monocytes, and inflammatory responses that start the induction of atherosclerosis. Atherosclerosis is hallmarked by the production of lipid-laden foam cells, particularly foam cells generated from macrophages, underneath the endothelial layer of the vascular wall. ApoE^-/-^ mice that had their blood flow disrupted experienced XBP1 splicing and persistent activation, which ultimately resulted in the apoptosis of endothelial cells (ECs) and the development of atherosclerotic plaques [64]. Zeng et al. [58] showed a correlation between EC proliferation and the transient activation of XBP1 splicing and found that persistent activation causes apoptosis of endothelial cells, loss of cells from vessel walls, and the formation of atherosclerotic plaques in an aorta isograft model by down-modulating VE-cadherin expression. This study also indicated that VE-cadherin expression can be downmodulated by XBP1s through transcriptional suppression and MMP-induced degradation. Emerging paradigms have revealed several roles of B cells that do not rely on antibodies and these functions may be significant in the onset and progression of atherosclerotic plaques. Andrew et al. [18] demonstrated that the loss of XBP1s leads to severe attenuation of the secretory capacity of plasma cells, which is enough to considerably enhance plaque formation and create a highly unstable plaque state in Ldlr^-/-^/XBP1s^B-cKO^ mice. Furthermore, XBP1s has been reported to be highly expressed in atherosclerosis and could boost macrophage survival and autophagy.

Recently, microRNAs (miRNAs) and long non-coding RNAs (lncRNAs) have been shown to perform a critical function in diverse biological and pathological activities in atherosclerosis. Meanwhile, previous research has proven that XBP1s, a signal transducer, contributes to atherosclerosis development by activating the apoptosis of ECs [64]. LINC00299 can work as a miR-135a-5p sponge to enhance XBP1s expression, thereby promoting the development of atherosclerosis [15].

## 5. The Role of XBP1s in Hypertension

Hypertension is among the most significant risk variables for CVDs. The accumulation of reactive oxygen species (ROS) derived from NADPH oxidase (Nox) leads to the dysregulation of ECs and consequent vascular injury, which are important elements in the pathophysiology of hypertension [65]. Livia et al. [66] showed that the IRE1–XBP1s pathway participates in Nox/ROS-regulated mechanisms, contributing to vascular dysfunction in hypertension. Furthermore, the pathophysiology of hypertension is linked to the activation of the renin angiotensin aldosterone system (RAAS) [67]. With regard to the role of XBP1s, research indicates that XBP1s binds to a site within the promoter region of ACE/ANGII/AT_1_R axis components. Consequently, this results in the degradation of vascular function, which eventually contributes to the occurrence of hypertension in rats [68]. It has been discovered that activation of HIF1a is a necessary step before XBP1s can bind to the corresponding response elements inside the promoter regions of the target genes in the RAAS [69]. Thus, IRE1–XBP1s, as an arm of the ER stress response, is involved in the signaling pathways of hypertension.

## 6. The Role of XBP1s in Cardiac Hypertrophy

**Figure 4:**
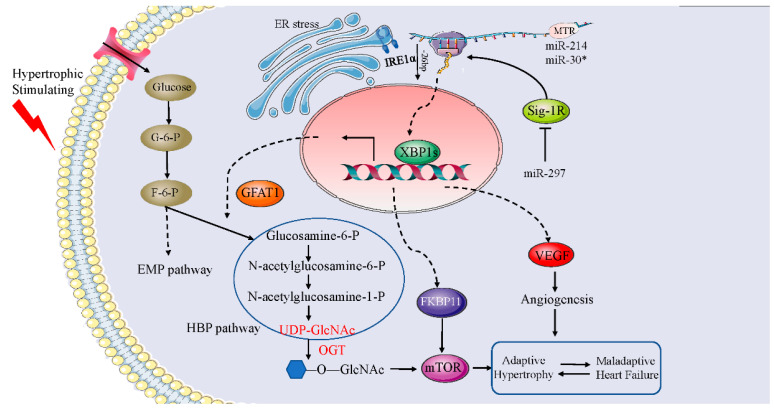
Schematic representation of the critical molecular mechanism of XBP1s in cardiac hypertrophy. XBP1s is a direct upstream transcriptional activator of multiple enzymes in HBP, one of which is called GFAT1. Under the action of GFAT1, glucose metabolism is shifted to the hexosamine biosynthesis pathway, thereby participating in the pathological process of cardiac hypertrophy through the mTOR pathway. Likewise, a unique transcriptional target of XBP1s, FKBP11 is involved in the mTOR pathway. MiR-297 upregulates the activation of XBP1s signaling by inhibiting the expression of the sigma-1 receptor (Sig-1R), thereby promoting cardiac hypertrophy. miR-30* and miR-214 target XBP1s to synergistically regulate cardiac VEGF production and angiogenesis. GFAT1: glutamine: fructose-6-phosphate amidotransferase 1; FKBP11: fk506-binding protein 11; miR-30*: miR-30 family.

Cardiac hypertrophy is an independent risk variable of cardiac-associated morbidity and mortality that develops as a consequence of cardiac overload, as well as the hyperactivation of neurohumoral systems and the presence of toxic metabolic compounds. Activation of XBP1s is one of the many complicated biomolecular processes that are involved in cardiomyocyte hypertrophy, which is a cellular reprogramming mechanism that integrates numerous other biomolecular pathways (Figure 4).

One of the most significant risk variables that might lead to cardiac hypertrophy is hypertension. When exposed to situations that cause hemodynamic stress, cardiomyocytes respond by inducing ventricular thickening and subsequent ventricular enlargement to alleviate wall stress. However, under persistent stress, this once adaptive response may become inefficient, resulting in heart failure [70]. There is a substantial body of research suggesting that metabolic dysregulation is among the most fundamental and early mechanisms underlying abnormal cardiac remodeling as a consequence of hypertension [71]. The hexosamine biosynthetic pathway (HBP), one of the routes by which glucose is metabolized, is maladaptive to pressure overload and thus contributes to pathological cardiac remodeling. Simultaneously, the persistent induction of glutamine: fructose-6-phosphate amidotransferase 1 (Gfat1), the rate-limiting enzyme of HBP, in the heart may directly activate the mechanistic target of rapamycin (mTOR) signaling, which acts as a trigger for pathological cardiac hypertrophy under hemodynamic stress. Moreover, XBP1s acts as a direct upstream transcription activator of a variety of enzymes that are part of the HBP, one of which is Gfat1 [72].

Cardiomyocytes may keep the heart functioning normally in cases where cardiac hypertrophy develops and are involved in the early adaptative phase. New research data strongly indicate that during the adaptive phase, XBP1s confers significant cardioprotection to prevent the progression of cardiac hypertrophy to heart failure. The process of cardiac remodeling includes metabolic dysregulation, hypoxia, changes in calcium signaling, and an increased demand for the synthesis of proteins. mTOR is a signaling nexus that is involved in both the sensing of nutrients and the modulation of metabolic processes. It also performs a fundamental function in hypertrophic growth that occurs as a result of pressure overload. A recent study suggests that activation of the mTOR signaling pathway is the mechanism via which XBP1s induces adaptive cardiac growth, and a unique transcriptional target of XBP1s, FK506-binding protein 11 (FKBP11), is responsible for mediating this process. In addition, knockdown of FKBP11 results in a considerable reduction in the rate of XBP1s-mediated mTOR activation and adaptive cell proliferation [73]. Another study came to the significant conclusion that XBP1s promotes cardiac hypertrophy triggered by NADPH oxidase 4 (NOX4) by activating RIPK1-related NF-κB signaling [74]. Further investigation revealed that insufficient angiogenesis is a crucial process in the progression of cardiac hypertrophy to heart failure. These results indicate that in the beginning stages of cardiac hypertrophy, an aberrant elevation in XBP1s levels is critical for the maintenance of VEGF-elicited cardiac angiogenesis, whose presence is responsible for the development of adaptive hypertrophy [17].

miRNAs are small non-coding RNAs that negatively regulate gene expression levels once they bind to the 3′ untranslated region (UTR) of the gene that is being targeted. Numerous studies have shown that miRNAs are involved in developing cardiac hypertrophy [75]. MiR-297 has been illustrated to upmodulate the activation of XBP1s signaling to promote cardiac hypertrophy by suppressing the expression of the sigma-1receptor (Sig-1R) [76]. Another study showed that during the transition from adaptive hypertrophy to heart failure, miR-30* and miR-214 target XBP1s to produce a synergistic effect that modulates cardiac VEGF production and angiogenesis [77].

These studies indicate that the advancement of cardiac dysfunction may be prevented by overexpressing XBP1s in a manner that is restricted to the heart. Thus, discovering the specific molecular processes through which XBP1s-elicited cardioprotection works may be helpful in the creation of innovative treatment techniques for the prevention or reversal of pathological hypertrophy.

## 7. The Role of XBP1s in Heart Failure

Heart failure is a diversified clinical syndrome resulting from cardiac overload and injury that leads to substantial morbidity and mortality. It can be classified into distinct phenotypes based on the volume of left ventricular ejection fraction (LVEF): heart failure with preserved ejection fraction (HFpEF), heart failure with mildly reduced ejection fraction (HFmrEF), or heart failure with reduced ejection fraction (HFrEF) [78]. Mizuho et al. [79] demonstrated that the XBP1s level was decreased in the impaired diastolic dysfunction group in contrast with that in the normal group via proteome analysis of human autopsy myocardium. XBP1s is a powerful transcriptional factor that acts via a range of transcriptional targets involved in several key cellular stress responses [80].

**Figure 5:**
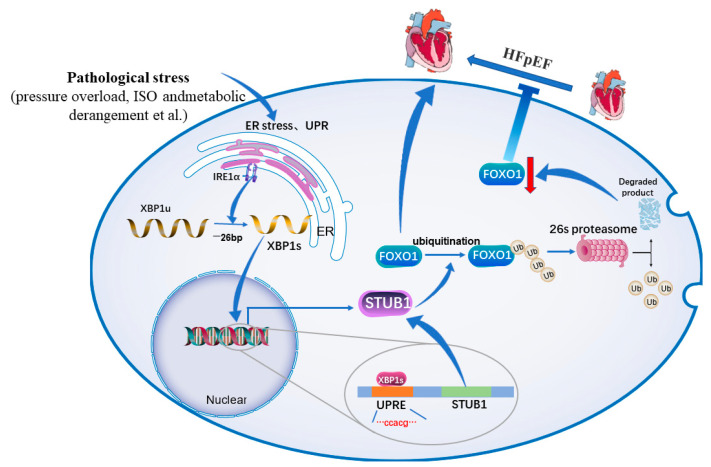
Schematic representation of the critical molecular mechanism of XBP1s in heart failure. Endoplasmic reticulum stress occurs in normal cardiomyocytes, XBP1s promotes the transcription of E3 ubiquitin ligase STUB1 by binding to the UPRE domain and promotes the ubiquitinated proteasomal degradation of FOXO1, thereby inhibiting the progression of heart failure. Under long-term pathological stimulation, endoplasmic reticulum stress is inhibited, XBP1 splicing is reduced, and FOXO1 is increased, promoting the progression of heart failure. FOXO1: Forkhead box protein O1, STUB1: STIP1 homology and U−Box−containing protein 1, ER: endoplasmic reticulum, UPR: unfolded protein response.

Recent research has shown that XBP1s can trigger promoter activity of brain natriuretic peptides (BNPs) by binding to an AP1/CRE-like element in the BNP promoter region. BNPs perform a momentous role in maintaining fluid homeostasis and cardiovascular growth. Thus, the induction of BNPs by XBP1s is indirectly conducive to the amelioration of cardiac dysfunction under inducible conditions [60]. Gabriele et al. [81] demonstrated that with a decrease in the abundance of XBP1s in mice with HFpEF, both diastolic dysfunction and symptoms of heart failure were reduced, suggesting that XBP1s in cardiomyocytes considerably drives HFpEF pathogenesis. Meanwhile, it was discovered that both experimental and clinical HFpEF exhibited an increase in the abundance of iNOS present in the myocardium. An increase in nitrosative stress and iNOS activity may enhance the S-nitrosylation of cysteine residues across numerous proteins, which disrupts their function. Furthermore, iNOS is responsible for modulating the activity of IRE1α by enhancing S-nitrosylation of IRE1, which ultimately leads to a decrease in the synthesis of XBP1s in cardiac myocytes. Past studies have demonstrated that metabolic derangement is decisive in the pathophysiological development of HFpEF based on both clinical trials and preclinical models. In the meantime, in mice, the expression of XBP1s, which is suppressed in HFpEF cardiac myocytes, attenuates the accumulation of lipids in the myocardium. Gabriele et al. also established that FOXO1 (Forkhead box protein O1) mediates lipid aggregation in cardiac myocytes, and up-modulation of XBP1s stimulates FOXO1 proteasomal degradation, as well as ubiquitination. An increased abundance and activity of FOXO1 contribute to increased myocardial lipid accumulation and cardiac metabolic modifications in HFpEF. The study also pointed out that in cardiac myocytes, the E3 ubiquitin ligase STIP1 homology and U-Box-containing protein 1 (STUB1), which is also a direct transcription target of XBP1s, performs an indispensable function in the degradation of FOXO1 that is dependent on XBP1s [38] (Figure 5). Doxorubicin is often limited in clinical application owing to its cardiotoxicity. Research has shown that doxorubicin can disturb ER function by generating ROS and disturbing Ca^2+^ homeostasis. XBP1s overexpression is involved in the attenuation of caspase-12 cleavage and doxorubicin-induced cardiomyocyte death [82]. Collectively, it appears that XBP1s has an integral function in the initiation and progression of heart failure.

## 8. Therapeutic Potential of XBP1s in Cardiovascular Diseases

Pathophysiological factors occurring in CVDs, such as metabolic disarrangements, hypoxia, and inflammation, can impose high demands on the ER protein-folding machinery, thereby triggering ER stress. In turn, ER stress induces inflammation and oxidative stress, the maladaptive UPR induces apoptosis and disruption of communication between the ER and other organelles in cardiomyocytes, and endothelial cells can exert negative effects on CVDs. Given the vital role of ER stress in the pathogenesis of CVDs, strategies targeting ER stress, particularly the maladaptive UPR, are emerging as therapeutic avenues for disease intervention. Many classic drugs used for the treatment of CVDs, such as statins, can modulate ER stress, although these might induce severe adverse effects. To this end, selectively and efficiently targeting ER stress holds promise for preventing and treating CVDs. As one of the core proteins of ERS and UPR, XBP1s plays a crucial role in cardiovascular diseases. It has been shown that increasing the levels of XBP1s by the use of AAVs is protective in various experimental disease settings. Hence, targeting XBP1s offers a promising treatment strategy for cardiovascular diseases. With the emergence of more mechanistic data, regulation of this pathway through its inhibition or activation, depending on the condition of the disease, may help achieve different clinical benefits.

Ginkgolide K significantly diminished the infarction size and improved heart function with decreased cardiomyocytes apoptosis in vivo models. In the meantime, activation of the IRE1α arm of the UPR, as shown by the increased level of phosphorylated IRE1α and XBP1 mRNA splicing, contributes to the elevation in the expression of XBP1s protein, which is a transcription factor for target genes, including GRP78 [83]. Studies have proven that restoration of XBP1s levels in failing cardiomyocytes results in diminished heart dysfunction and amelioration of the heart failure phenotype [81].

Imeglimin hydrochloride is a first-in-class glimin for treating type 2 diabetes [84]. In a HFpEF model, imeglimin inhibited iNOS upmodulation while simultaneously restoring the XBP1s and STUB1 expression, which is a significant contributor to the degradation of FoxO1, an immediate target of XBP1′s transcriptional activity, located downstream of XBP1s and is implicated in the formation of HFpEF and the progression of cardiac adipogenesis. Cardiomyocyte-specific overexpression of XBP1s in HFpEF mice can improve the HFpEF phenotype. Similarly, imeglimin normalizes the downregulation of IRE1a-XBP1 signaling concurrently with reducing the phenotype of HFpEF [85].

In recent years, some new agonists or stabilizers of XBP1s have been found and improved. For example, synthesized (Z)-*N*-dihydrocoptisine-8-ylidene aromatic amines, dihydrocoptisine, (±)-8-acetyl dihydrocoptisine of QCA derivatives, and HLJ2 have been ascertained to show significant activation of XBP1s transcription activity. These new compounds may soon be used in more cardiovascular research and clinical applications [86,87,88].

## 9. Conclusions and Future Directions

XBP1s functions as a key mediator of the ER stress response. Recent studies indicate that XBP1s may have a function in the pathogenic mechanisms that underlie various CVDs, including VSMC dysfunction, oxidative stress, and inflammation. To conclude, XBP1s is strongly linked to CVDs and has a role in the onset and progression of CVDs. XBP1s is a possible treatment targets for CVDs. Nonetheless, the mechanisms of XBP1s in CVDs have not yet been explored comprehensively. As a consequence, additional experimental data and clinical samples are required to thoroughly assess the link between XBP1s and cardiovascular disorders.

## Figures and Tables

**Table 1:** Cellular functions of XBP1s and related mechanism of action.

Function	Mechanism	Ref.
Transcriptional Regulation	By binding to the UPRE of the Krüppel-like factor 9 (KLF9) promoter, XBP1s up-modulates the KLF9 transcription in the case of severe ER stress.	[19]
	XBP1s induces ER expansion by activating the synthesis of phosphatidylcholine (PtdCho).	[20]
	XBP1s can upregulate insulin-like growth factor binding protein-3 (IGFBP3) expression.	[21]
	Upon ER stress, XBP1s specifically induces Forkhead Box A3 (FOXA3). FOXA3 exacerbates the excessive lipid accumulation induced by the acute ER-inducer TM.	[22]
	XBP1s binds to the XBP1-binding site in the Forkhead Box A2(FOXA2) promoter.	[23]
	XBP-1s may directly upregulate Kaposi’s Sarcoma-Associated Herpesvirus-Encoded Thymidine Kinase (ORF21).	[24]
	The transcriptional activity of E1A is increased as a result of the binding of XBP1s to the E1A enhancer/promoter.	[25]
	XBP1s is capable of binding to and recruiting RNA polymerase II to the IL6, SNAI1, and MMP9 promoters, and the intragenic super-enhancer of glutamine-fructose-6-phosphate transaminase 2 (GFPT2).	[26]
	XBP1s activates MYC proto-oncogene expression.	[27]
	UDP-galactose-4-epimerase (GalE), a direct target of XBP1′s transcriptional function, is a new modulatory nexus that connects the UPR to the characteristic postprandial metabolic alterations in hepatocytes.	[28]
	The activity of the histone acetyltransferase p300 contributes to an increase in the stabilization of the spliced form of X-box binding protein 1 (XBP1s) and stimulates the transcription of XBP1′s target gene known as homocysteine inducible endoplasmic reticulum protein with ubiquitin-like domain 1 (Herpud1).	[29]
Insulin Signaling	IGF-1 is responsible for inducing ER biogenesis in the bovine MEC line via the activation of the IRE1-XBP1 axis following the modulation by mTORC1.	[30]
Inflammation	Icariin (ICA) may reduce the expression level of TNF-α, IL-6, and IL-1β, by blocking the IRE1/XBP1s pathway.	[31]
	The ER stress-inducing agent tunicamycin (Tm) and PA treatments significantly activate the IRE1α-XBP1s signaling pathway and increase the expression of pro-inflammatory mediators, including TNF-α, IL6, and IL1β.	[32]
	LncRNA H19 plays a vital function in modulating inflammation in retinal endothelial cells when subjected to high-glucose conditions via the modulation of the miR-93/XBP1s axis.	[33]
	The activation of the triggering receptor expressed on the myeloid cells 1 (TREM-1) pathway in macrophages causes ER stress to occur via the IRE-1α/XBP-1s pathway, thus contributing to the pro-inflammatory milieu.	[34]
Oxidative stress	Complete activation of UPR, which would alleviate ER stress, is associated with fructose-induced oxidative stress.	[35]
Autophagy	Under PD conditions, the functional loop that governs mitophagy between XBP1s and PINK1 was disturbed.	[36]
	ER stress/UPR is a mechanism that ultimately results in the activation of caspase-induced proteolysis and an elevation in the level of expression of genes associated with autophagy.	[37]
Metabolism	XBP1s overexpression in cardiac myocytes improves the HFpEF phenotype in mice and decreases the accumulation of lipids in the myocardium.	[38]
	XBP1s may up-modulate the expression of particular enzymes implicated in the metabolism of glucose and eventually enhance the O-linked β-N-acetylglucosamine modification (O-GlcNAcylation).	[39]
Apoptosis	Inhibiting XBP1s expression might substantially improve the activities of lactate dehydrogenase and creatine kinase-MB, as well as cell apoptosis, and as a result, activated ischemia/reperfusion-mediated H9c2 cell damage is exacerbated.	[40]
	Zonisamide (ZNS) up-modulated Bcl-2 activity, inhibited Bax and caspase-3 activity, reduced the number of TUNEL-positive cells in cardiac tissues, and protected against cardiac hypertrophy induced by type 2 diabetes by reducing the rate of apoptosis caused by ER stress (IRE-1α/XBP-1s).	[41]
	Grape seed proanthocyanidin extract (GSPE) relieved ER stress-elicited apoptosis via suppressing the IRE1α/XBP-1S/caspase-12 and PREK/eIF2α pathways.	[42]
	IRE1 made a cell-type-specific contribution to the Tg-elicited cell death. This was linked to the stimulation of c-Jun N-terminal kinase that was reliant on XBP1s, whose impact was pro-apoptotic in LNCaP cells but had no impact on HCT116 cells.	[43]
	STF-083010 reduced cell proliferation and induced apoptosis through XBP1/CHOP/Bim signaling pathway.	[44]
Hypoxia	The stimulation of the IRE1α/XBP1s/ HIF-1α pathway was considerably inhibited when HSP47 was silenced by the use of small interfering RNA.	[45]
	The activation of p300 by hypoxia amplifies the unfolded protein response (UPR) that is mediated by XBP-1s, helps prevent the degradation of XBP1s that is reliant on the proteasome, and increases the level of transcriptional activity carried out by XBP1s for Herpud1.	[29]
	The activity of IRE1 in response to hypoxia elevates the levels of the HIF1α protein in a way that is independent on XBP1s.	[46]
Angiogenesis	The expression of XBP1s enhances cell proliferation, migration, and angiogenesis, which helps to reverse the damage caused by miR-33a-5p.	[47]
	In BMECs that had been treated with OGD, XBP1s overexpression led to an increase in the expression of HIF1α, VEGF, p-extracellular signal-regulated kinase1/2, p-GSK3β, p-mTOR, p-AKT, and phosphatidylinositol-4,5-bisphosphate 3-kinase.	[48]
	Through the activation of XBP1s, the ER stress activator tunicamycin enhanced the production of VEGFA that was produced in human granulosa cells as a result of human chorionic gonadotropin induction. VEGFA performs a fundamental function in ovarian angiogenesis.	[49]
	XBP1s is responsible for the modulation of VEGF-induced angiogenesis in the heart and has a role in the development of adaptive hypertrophy.	[17]

## Data Availability

Not applicable.

## Abbreviations

XBP1s	Spliced X-box binding protein-1
CVDs	Cardiovascular diseases
ERS	Endoplasmic reticulum stress
UPR	Unfolded protein responses
ERAD	ER-associated degradation
IRE1a	Inositol-requiring kinase 1
ATF6	Activating transcription factor 6
PERK	Protein kinase-like ER kinase
eIF2α	Eukaryotic translation initiation factor 2α
ATF4	Activating transcription factor 4
CREB	cAMP-response element-binding
bZIP	Basic-region leucine zipper
SMCs	Smooth muscle cells
VEGF	Vascular endothelial cell growth factor
PDGF	Platelet-derived growth factor
bFGF	Basic fibroblast growth factor
CNN1	Calponin h1
COL4A1	Collagen alpha 1
VPCs	Vascular progenitor cells
TG2	Transglutaminase 2
TLR	Toll-like receptors
ROS	Reactive oxygen species
RAAS	Renin-angiotensin-aldosterone system
HBP	Hexosamine biosynthetic pathway
Gfat1	Glutamine: fructose-6-phosphate amidotransferase 1
mTOR	Mechanistic target of rapamycin
FKBP11	FK506-binding protein 11
NOX4	NADPH oxidase 4
Sig-1R	Sigma-1receptor
HFpEF	Heart failure with preserved ejection fraction
HFmrEF	Heart failure with mildly reduced ejection fraction
HFrEF	Heart failure with reduced ejection fraction
LVEF	Left ventricular ejection fraction
BNP	Brain natriuretic peptide
FOXO1	Forkhead box protein O1
STUB1	STIP1 homology and U-Box-containing protein 1
